# Community Engagement in Long Covid Research: Process, Evaluation and Recommendations From the Long COVID and Episodic Disability Study

**DOI:** 10.1111/hex.70365

**Published:** 2025-08-10

**Authors:** Margaret E. O'Hara, Kiera McDuff, Hannah Wei, Lisa McCorkell, Catherine Thomson, Mary Kelly, Susie Goulding, Imelda O'Donovan, Sarah O'Connell, Ruth Stokes, Nisa Malli, Natalie St. Clair‐Sullivan, Soo Chan Carusone, Angela M. Cheung, Kristine M. Erlandson, Ciaran Bannan, Liam Townsend, Colm Bergin, Jaime H. Vera, Richard Harding, Lisa Avery, Darren A. Brown, Kelly K. O'Brien

**Affiliations:** ^1^ Long Covid Support London UK; ^2^ Department of Physical Therapy, Temerty Faculty of Medicine University of Toronto Toronto Canada; ^3^ Patient‐Led Research Collaborative Ottawa Canada; ^4^ Patient‐Led Research Collaborative Oakland California USA; ^5^ Long COVID Physio London UK; ^6^ COVID Long‐Haulers Support Group Canada Toronto Canada; ^7^ Long COVID Advocacy Ireland; ^8^ Patient‐Led Research Collaborative Toronto Canada; ^9^ Department of Global Health and Infection, Brighton and Sussex Medical School University of Sussex Brighton UK; ^10^ Royal Sussex Hospital Brighton and Sussex University Hospitals NHS Trust Brighton UK; ^11^ Cicely Saunders Institute of Palliative Care, Policy & Rehabilitation King's College London London UK; ^12^ McMaster Collaborative for Health and Aging Hamilton Canada; ^13^ Institute of Health Policy, Management and Evaluation (IHPME), Dalla Lana School of Public Health University of Toronto Toronto Canada; ^14^ Department of Medicine University of Toronto Toronto Canada; ^15^ Department of Medicine University Health Network Toronto Canada; ^16^ Deptartment of Medicine University of Colorado Anschutz Medical Campus Colorado USA; ^17^ Department of Infectious Diseases St James's Hospital Dublin Ireland; ^18^ Department of Clinical Medicine Trinity College Dublin Dublin Ireland; ^19^ Biostatistics Department, Princess Margaret Cancer Centre University Health Network Toronto Canada; ^20^ Dalla Lana School of Public Health University of Toronto Toronto Canada; ^21^ Chelsea and Westminster Hospital NHS Foundation Trust London UK; ^22^ Rehabilitation Sciences Institute (RSI) University of Toronto Toronto Canada

**Keywords:** community engagement, episodic disability, Long Covid, patient and public engagement, post‐Covid condition

## Abstract

**Introduction:**

Long Covid and other infection‐associated chronic condition communities have been integral in advocating for patient engagement in all stages of research, from design and conduct, and implementation, through to interpretation and knowledge translation; nevertheless, the process varies across research teams. In this paper, we (1) describe our process undertaking a community‐engaged Long Covid research study; (2) evaluate our community‐engaged approach, highlighting strengths and limitations with our process; and (3) identify recommendations for engaging in community‐engaged patient‐oriented research in Long Covid.

**Methods:**

Guided by the 4PI (Principles; Purpose; Presence; Process; Impact) Framework and Patient‐Led Research Scorecards, we describe our community‐engaged approach within the Long COVID and Episodic Disability Study, followed by an evaluation of our community engagement using a multistage consultation with members of the Long COVID and Episodic Disability Study team. We conducted an online group‐based discussion among persons with lived experiences and administered a web‐based Scorecard questionnaire rating the collaboration as it relates to four domains of patient burden, governance, integration into the research process, and organisation readiness to all members of the team, to assess strengths and limitations of our approach. Scores ranged from −2 (non‐collaboration) to +2 (ideal collaboration).

**Results:**

Ten team members, five of whom were persons with lived experiences, completed the Scorecard questionnaire. Median Scorecard scores ranged from +1 to +2 for all domains. Five team members with lived experiences, representing four community support groups and organisations that participated in the community‐engagement discussion. We describe the practices and principles that enabled meaningful community engagement, with the strengths and limitations of our approach embedded throughout.

**Conclusion:**

Our community‐engaged approach to the Long COVID and Episodic Disability Study enhanced the quality and relevance of the study to the community while highlighting areas to heighten meaningful engagement throughout. This study builds on foundational community‐based research principles of patient‐oriented research. Recommendations derived from our experiences may be used by other research teams conducting community‐engaged patient‐oriented research.

**Patient or Public Contribution:**

The Long COVID and Episodic Disability Study is a community‐engaged research study involving 25 members, including 12 persons living with long Covid, 13 researchers and 5 clinicians (categories are not mutually exclusive), referred to as the Full Team. Persons with lived experiences possessed a range of professional and personal experiences spanning research, clinical, policy and private sector/business contexts; team members wore multiple hats and perspectives which collectively strengthened the diversity of expertise, perspectives and insights to the team and process. Engagement of people with lived experiences with Long Covid ensured that the study was fully co‐created with people living with Long Covid. During the development of the study proposal, community partners from organisations in Canada, Ireland, the United Kingdom and the United States, who were linked to larger networks of people living with Long Covid, were purposefully invited to join the study team. Several Long Covid community networks and organisations, represented by persons living with Long Covid, were involved in all stages of the research, including: COVID Long‐Haulers Support Group Canada (S.G.); Long COVID Advocacy Ireland (I.O., S.O. and R.S.); Long COVID Ireland (N.R. and R.S.); Long COVID Physio (D.A.B. and C.T.); Long Covid Support UK (M.O.H.); and Patient‐Led Research Collaborative (L.M., N.M. and H.W.). These representatives along with the Co‐PIs (K.K.O. and D.A.B.) and co‐ordinator (K.M.) comprised the Core Long COVID and Episodic Disability Community Collaborator Team (Core Team). Team members with lived experiences were provided yearly remuneration for their time and expertise dedicated to the study, either as an individual, or to the community organisation which they represented on the study according to their preference.

## 1 Introduction

### 1.1 Overview of Community Engagement in Research

Community engagement in research is critical to ensure relevant, meaningful and impactful research that will advance clinical practice and policy for persons living with complex chronic conditions. Community engagement, also known as Patient and Public Involvement (PPI), is described as research done ‘with’ or ‘by’ people with lived experience, rather than ‘to’, ‘about’ or ‘for’ them [1, 2], or ‘meaningful and active collaboration’ [3]. People with lived experiences are critical to the research process as they bring specialised knowledge of health conditions, and their input improves the quality and relevance of research [4]. Actively involving people with lived experiences of health conditions in the design and management of health policy, services and research has increased since the 1990s [4]. This growth was largely grounded within the activism of people with lived experiences of disability and illness, and community principles of participation, of ‘nothing about us without us’ [5, 6, 7]. For example, persons living with HIV developed position statements asserting their right to be involved in decisions about them, such as the Greater Involvement of People Living with HIV/AIDS (GIPA) and Meaningful Engagement of People Living with HIV/AIDS (MEPA) [8, 9, 10, 11]. Investigators should engage patients in all stages of research, from design and conduct, through to interpretation, dissemination and implementation [12]. Guidance documents exist to advise researchers embarking on patient engagement in research [13]. Some national funding organisations have established guidance and resources for fostering meaningful community engagement, such as the Canadian Institutes of Health Research (CIHR), Strategy for Patient‐Oriented Research (SPOR) and National Institute for Health Research (NIHR) PPI in research. These funders require investigators seeking their funding to follow this guidance [2, 3]. Despite the importance of community engagement in research and existing guidance, community engagement has yet to be fully embedded in all aspects of health research [14].

While a range of terminology exists to characterise PPI in research, we use ‘community engagement’ as the concept of inquiry and refer to team members with lived experiences as ‘community partners’ in this manuscript. We chose this terminology to reflect leadership among members with lived experiences on the team within the wider community of people with Long Covid.

### 1.2 Evolution and Importance of Community Engagement in Long Covid Research

The Covid‐19 pandemic highlighted the need for community advocacy and representation throughout the research process. Community engagement was particularly critical in the case of this newly emerging disease condition, and one which was overlooked by research and health authorities in the early days of its emergence. The voices of those with lived experiences with Long Covid have been instrumental in driving research on this condition. Early in the Covid‐19 pandemic, many were noticing that they were experiencing symptoms of Covid‐19 for much longer than the acute period and recognised these symptoms as a unique manifestation of Covid‐19. As early as April 2020, people with lingering illness from SARS‐CoV‐2 had self‐organised into online groups and were sharing their experiences [15, 16]. The term Long Covid was coined by a prominent patient advocate and taken up by the community [15]. A group of people with Long Covid founded the Patient‐Led Research Collaborative (PLRC) and were the first to document the multiple symptoms associated with Long Covid on 6 May 2020 [17, 18], which were then subsequently expanded on in a survey documenting over 200 symptoms. Since then, there has been an explosion of research seeking to define and characterise Long Covid [19, 20, 21, 22, 23], identify potential causes of this condition [24], and explore potential treatments [25]. Despite community leadership in Long Covid research, persons with lived experiences are not always involved throughout the research process from inception to dissemination, nor meaningfully engaged in the process [26]. Tokenism, (unconscious) undervaluing of lived experiences, under‐investing in support for patient partners (e.g., lack of or insufficient compensation for patient partners), and under‐estimating the vulnerability of patient partners persist, highlighting gaps in community engagement in research [26].

### 1.3 Introducing Long COVID and Episodic Disability Study

The Long COVID and Episodic Disability Study aims to characterise and measure disability experienced among people living with Long Covid [27]. This international study was conceived by a member of the team (D.B.) who has lived experiences of Long Covid and was instrumental in the creation of the patient‐led organisation, Long COVID Physio [28]. As a physiotherapist with specialisation in HIV, his perspectives allowed him to understand that, like HIV, Long Covid can be understood as an episodic illness and that lessons learned from the conceptual Episodic Disability Framework could be usefully modified for Long Covid [29, 30].

Despite the existence of guidance documents and toolkits [13, 31], it is still possible for researchers to follow all the advice and ‘do’ apparent community engagement while being tokenistic [14, 32]. We do not wish to repeat the excellent practical advice which can already be found in numerous publications [33, 34, 35, 36, 37, 38]. In this paper, we aim to offer insights beyond the process elements of community engagement and instead elucidate the practices which allow research teams to move beyond tokenism. Specifically, we aim to: (1) describe our process of undertaking a community‐engaged Long Covid research study (methods); (2) evaluate our community‐engaged approach, highlighting strengths and limitations with our process (results); and (3) identify recommendations for engaging in community‐engaged patient‐oriented research in Long Covid (discussion).

## 2 Materials and Methods

We conducted a multi‐stage consultation with members of the Long COVID and Episodic Disability Study team, including persons with lived experiences, researchers, clinicians and staff about their experiences collaborating on the study. In the methods, we describe: (a) our community‐engaged approach within the exemplar of the Long COVID and Episodic Disability Study, followed by (b) our consultation process to assess strengths and limitations of our approach.

a. The Context and Exemplar—Long COVID and Episodic Disability Study

Funded by the CIHR, the aims of the Long COVID and Episodic Disability Study are: (1) to characterise disability experiences among people living with Long Covid in Canada, Ireland, the United Kingdom and the United States and (2) to develop and assess the measurement properties of a patient‐reported outcome measure (PROM) to describe the presence, severity and episodic nature of Long Covid [27]. Aim 1 has been addressed [39, 40], and Aim 2 is currently in progress. For Aim 1, we conducted a community‐engaged qualitative descriptive study involving online one‐on‐one semi‐structured interviews with 40 adults living with Long Covid in the four countries. Participants were asked to describe their health challenges living with Long Covid and the impact on their overall health [27, 39]. For Aim 2, we used findings from the interviews to refine an existing generic disability questionnaire for use with adults living with Long Covid [27]. We conducted a measurement study involving a web‐based survey whereby we administered the Episodic Disability Questionnaire (EDQ) to determine its measurement properties to describe the presence, severity and episodic nature of disability among adults living with Long Covid.

The Long COVID and Episodic Disability Study team is co‐led by a person with lived experiences of Long Covid in the United Kingdom (D.B.) and a researcher in Canada (K.K.O.), derived from foundational work and collaborations between K.K.O. and D.B. on episodic disability and rehabilitation in the context of HIV (Episodic Disability Framework) [41, 42]. D.B. and K.K.O. identified potential similarities among persons with Long Covid and the uncertain and potentially episodic nature of disability associated with Long Covid [29, 30]. The Long COVID and Episodic Disability Study was approved by the Health Sciences Research Ethics Board (REB) at the University of Toronto (Protocol #41749) and the Saint James Hospital (SJH)/Tallaght University Hospital (TUH) Joint Research Ethics Committee (2024‐Mar‐34453445). Details of the study protocol have been previously published [27].

### 2.1 Involvement of Persons With Lived Experiences on the Team

The Long COVID and Episodic Disability Study is a community‐engaged research study involving 25 members, including 12 persons living with Long Covid, 13 researchers and 5 clinicians (categories are not mutually exclusive), referred to as the Full Team. Persons with lived experiences possessed a range of professional and personal experiences spanning research, clinical, policy and private sector/business contexts; team members wore multiple hats and perspectives which collectively strengthened the diversity of expertise, perspectives and insights to the team and process. Engagement of people with lived experiences with Long Covid ensured that the study was fully co‐created with people living with Long Covid. During the development of the study proposal, community partners from organisations in Canada, Ireland, the United Kingdom and the United States, who were linked to larger networks of people living with Long Covid, were purposefully invited to join the study team. Several Long Covid community networks and organisations, represented by persons living with Long Covid, were involved in all stages of the research, including: COVID Long‐Haulers Support Group Canada (S.G.) [43]; Long COVID Advocacy Ireland (I.O., S.O. and R.S.) [44]; Long COVID Ireland (N.R. and R.S.); Long COVID Physio (D.A.B. and C.T.) [45]; Long Covid Support UK (M.O.H.) [46]; and PLRC (L.M., N.M. and H.W.) [47]. These representatives, along with the Co‐PIs (K.K.O. and D.A.B.) and co‐ordinator (K.M.), comprised the Core Long COVID and Episodic Disability Community Collaborator Team (Core Team). Team members with lived experiences were provided yearly remuneration for their time and expertise dedicated to the study, either as an individual, or to the community organisation which they represented on the study according to their preference.

### 2.2 Team Process, Roles and Communications

The Core Team guided all stages of the research study. The Core Team was comprised of Co‐PIs (K.K.O. and D.A.B.), persons with lived experiences representing the above community networks (S.G., I.O., S.O., R.S., N.R., R.S., C.T., M.O.H., L.M., N.M. and H.W.), and clinicians and researchers from St. James's Hospital (C.B., C.Ba. and L.T.). The Core Team was responsible for recruitment, refinement of data collection tools, interpretation of findings, and decisions surrounding ethical considerations as they related to safe engagement with persons with Long Covid and knowledge translation. The Core Team worked closely with the broader research team at all stages of the research. The Core Team met frequently (monthly to bimonthly) and also met with the broader research team (Full Team) at milestones throughout.

Research staff used several channels for communication among the Full Team and Core Team, including email, online platforms (Zoom), and circulating written materials via attachments in email. Staff used email to schedule and send reminders about team meetings. Staff arranged meeting times to accommodate team members across different time zones. Staff circulated documents approximately 1 week in advance of (e.g., agenda, summaries of results and meeting minutes) or after (e.g., minutes) meetings. The co‐ordinator (K.M.) circulated same‐day reminders for each meeting to aid those with cognitive constraints. Staff provided regular email updates on the progress of the study, including upcoming meetings, status of recruitment and upcoming knowledge translation opportunities. Out of respect for the limited time and energy of those contributing, staff encouraged open feedback about the need for flexibility in terms of means of study engagement and timelines for task completion. When team members were unable to attend meetings, the research staff offered phone or online calls at a convenient time to follow up one‐on‐one with the co‐ordinator or study Co‐PIs. Staff provided generous lead times for completion of tasks. However, timelines were also made flexible based on the capacity and schedule of team members.

### 2.3 Recruitment Phases of Study

Team members with lived experiences of Long Covid and representatives of community organisations led the recruitment of adults living with Long Covid to the study. Community partners promoted the study widely within their networks. Recruitment was an iterative and purposeful process whereby the Core Team met weekly during phase 1 (interviews) to discuss the characteristics of participants to date in relation to our aim to achieve diversity in age, gender identity, ethnicity, sexual orientation and time since initial Covid‐19 infection. The Core Team continued to meet bi‐weekly during phase 2 (web‐based survey) during the recruitment phase to discuss the process of recruitment, geographical (country) representation and any issues with recruitment or data collection.

### 2.4 Refinement of Data Collection Tools and Analysis

Members of the Core Team were involved in refining the interview guide for phase 1 and developing and refining the PROMs for the web‐based questionnaire for phase 2. During phase 1 (interviews), the Core Team was involved in group‐based qualitative analysis of interview data using content analytical techniques [48]. The Core Team met twice to review coding and participant summaries for each interview and discussed interpretations of the analytical categories representing experiences of disability. After the interview analysis, the Core Team mapped categories of disability as experienced by adults living with Long Covid to the existing EDQ questions, originally derived from and with persons living with HIV [49, 50, 51, 52]. For phase 2, the team set out to identify a PROM to assess the presence, severity and episodic nature of disability among adults living with Long Covid. The Core Team met on two subsequent occasions to generate items for a supplemental questionnaire that represented categories of disability from the interviews that were not captured in the existing EDQ. The study team met twice (once as a Core Team and once as a Full Team) to review, prioritise and refine items, resulting in a draft Long COVID Supplement Questionnaire. The Full Team reviewed and refined the wording of the items and preamble to establish a Long COVID Episodic Disability Questionnaire (LC‐EDQ)—Supplement. The study team is currently assessing the measurement properties of the EDQ and LC‐EDQ Supplement.

### 2.5 Knowledge Translation

The Core Team is involved in co‐authoring presentations, publications (including study protocol) and other knowledge translation outputs from the Long COVID and Episodic Disability Study [27, 39, 40]. Manuscript writing involves a co‐creation approach. Community partners provide feedback on all manuscript drafts. Comments and amendments are synthesised by the study team. Feedback on manuscripts is provided via email, track changes in a Word document, or Zoom. Research staff are flexible with timelines on feedback to accommodate capacity and competing responsibilities of team members.

b. Evaluating Strengths and Limitations of Our Community‐Engaged Process

Authors (M.O.H., K.K.O. and K.M.) conducted a multi‐stage reflection with members of the Long COVID and Episodic Disability Study team (including community partners, researchers, clinicians and staff) regarding their experiences with community engagement in the study. We reached out to the REB, inquiring about our need for ethics review and approval. We received confirmation that, given this was an ongoing and iterative reflective process among members of the research team, we did not require REB approval for this evaluation.

At the end of each Core or Full Team meeting, team members were asked about their experiences and specifically asked to share their perspectives and feedback about the team process and what it was like to be involved in this study team.

Our evaluation was guided by a framework for describing patient engagement (4PI) [34] and Patient‐Led Research Scorecards [53] to identify strengths and challenges of community (or patient) engagement in the study. Evaluation of the effectiveness of patient engagement in research is not straightforward, and a number of impact evaluation frameworks exist [54, 55]. We chose to use the 4PI and Patient‐Led Research Scorecards as they are simple in their construction and easy to understand and were developed principally by people with lived experiences [34, 53]. The 4PI Framework is a commonly used framework for evaluation of PPI, and the lead author (M.O.H.) was familiar with its use [34, 53, 56]. Launched in 2013 and developed by the National Survivor User Network (NSUN), the 4PI Framework stands for ‘Principles; Purpose; Presence; Process; Impact’ [34]. This framework codifies ways of working between groups of people with a common purpose or project. The 4PI framework helps working groups think through their *purpose*, *how* they work together and *how* they will evaluate whether they have achieved their purpose. Examples of its use can be seen on the NSUN website [56].

The Patient‐Led Research Scorecards were published in 2023 as a patient‐centred, practical evaluation on the fairness and effectiveness of patient group and research partner collaboration [53]. Although the Scorecards are not specific to Long Covid, they were designed and written by the PLRC, including persons with lived experiences of Long Covid, and written with the needs of people with chronic health conditions in mind [53]. Some developers of the PLRC Scorecards are members of the Long COVID and Episodic Disability Study team and are co‐authors of this manuscript (H.W. and L.M.). We chose the Patient‐Led Scorecards because they were recently developed by persons with lived experiences with Long Covid. This was the first known study to use them in a formal evaluation of the community‐engaged research process. The 4PI framework is discursive, while the Scorecards provide numerical scores. Given the strengths of both, we used both to inform our approach.

### 2.6 Guided Discussion Using the 4PI Framework

In January 2024, the lead author and community partner (M.O.H.) met with other team members with lived experiences online (Zoom) to discuss the process and impact of patient engagement in the Long COVID and Episodic Disability Study. During this 90‐min discussion, community partners were asked to reflect on how they had worked together throughout the study according to the categories of the 4PI. MOH provided examples as a starter for discussion and then further populated the categories as the discussion ensued. M.O.H. asked team members to share their considerations for future patient engagement in Long Covid research. See Supplemental File 1 for the guiding questions in the discussion. M.O.H. recorded the meeting and took notes to capture the discussion. No researchers were present at this meeting to enable community members to speak freely.

### 2.7 Patient‐Led Research Scorecards

In March and April 2024, authors (M.O.H., K.K.O. and K.M.) transferred the contents of the PLRC Scorecards to an online questionnaire to make it easier to use and collect the data. This questionnaire was then sent to all members of the Long COVID and Episodic Disability Study team to obtain perspectives on patient engagement efforts during the study. Using the Scorecards as a foundation, authors asked all members of the team to reflect on and discuss their experiences with the study, either as an organisation or as an individual. The questionnaire included 14 items separated into the following four domains of the Scorecards: (1) Patient Burden; (2) Patient/Partner Governance; (3) Integration Into the Research Process; and (4) Research Organisation Readiness [53]. Team members were asked to rate their perception of patient engagement in the Long COVID and Episodic Disability Study team from non‐collaboration (−2) to ideal collaboration (+2). Each section included open‐ended text responses to allow for further expansion. The questionnaire also included two questions on the usefulness of the Scorecards to elicit perceptions on patient collaborative research efforts, with a range from −2 (not useful at all) to +2 (very useful) and asked about recommendations for how the Scorecards might be used to enhance collaborative engagement. M.O.H., K.K.O. and K.M. administered two versions of the questionnaire, one tailored to patient partners and the other tailored to researchers and clinicians on the team. See Supplemental File 2 for the questionnaire.

### 2.8 Developing Recommendations

We consolidated comments derived from the guided discussion and Patient‐Led Research Scorecards to develop recommendations for engaging in community‐engaged patient‐oriented research in Long Covid. The lead author (M.O.H.) triangulated these two data sources with the aim of deriving practical recommendations that can be applied across different community‐engaged and participatory research contexts. All members of the co‐authorship team reviewed and refined the recommendations.

## 3 Results

We describe the results from the Scorecard questionnaire, followed by the consultation and reflections on the strengths and limitations and the impact of our approach.

### 3.1 Patient‐Led Research Scorecard Questionnaire

Of the 24 team members of the Long COVID and Episodic Disability Study, 10 (42%) completed the self‐reported Scorecard questionnaire. Of those 10 team members, five (50%) were researchers and five (50%) were community partners. Roles of the researchers (*n* = 5) on the team were self‐identified as ‘researcher’ (*n* = 3/5; 60%) and ‘clinician’ (*n* = 2/5; 20%). Among the five community partners, four specified their role on the team; one (1/4; 25%) identified as a ‘researcher’ and three (3/4; 75%) as a ‘person with lived experiences’. Four of the five community partners (80%) completed the questionnaire as individuals, and one (20%) completed the questionnaire in collaboration with other members of the community network they represented.

Team members ranked their agreement with the statements for each of the four domains of the Scorecards. The median scores (range −2 to +2) across questions in the Patient Burden and Research Organisation Readiness domains were +1 among researchers and +2 among community partners. The median scores for Patient/Partner Governance were +1 among both researchers and community partners. The median score for integration into the research process was +2 among both researchers and community partners. See Table 1 for details.

**Table 1:** Responses to the Patient‐Led Research Scorecard Questionnaire.

Patient‐Led Research Scorecard Questionnaire Items		Researchers (*n* = 5) *N* (%)	Patient partners (*n* = 5) *N* (%)
Patient Burden	What statement best describes the nature of collaboration related to accessible engagement?		
	Don't know or not applicable	0 (0%)	0 (0%)
	Non‐collaboration (−2)	0 (0%)	0 (0%)
	Minimal collaboration (−1)	0 (0%)	0 (0%)
	Acceptable collaboration (0)	0 (0%)	1 (20%)
	Great collaboration (+1)	4 (80%)	1 (20%)
	Ideal collaboration (+2)	1 (20%)	3 (60%)
	What statement best describes the nature of collaboration related to trauma‐informed practices?		
	Don't know or not applicable	0 (0%)	0 (0%)
	Non‐collaboration (−2)	0 (0%)	0 (0%)
	Minimal collaboration (−1)	0 (0%)	0 (0%)
	Acceptable collaboration (0)	0 (0%)	2 (40%)
	Great collaboration (+1)	4 (80%)	1 (20%)
	Ideal collaboration (+2)	1 (20%)	2 (40%)
	What statement best describes the nature of collaboration related to responsiveness to patients?		
	Don't know or not applicable	0 (0%)	0 (0%)
	Non‐collaboration (−2)	0 (0%)	0 (0%)
	Minimal collaboration (−1)	0 (0%)	0 (0%)
	Acceptable collaboration (0)	0 (0%)	0 (0%)
	Great collaboration (+1)	4 (80%)	0 (0%)
	Ideal collaboration (+2)	1 (20%)	5 (100%)
	What statement best describes the nature of collaboration related to compensation?		
	Don't know or not applicable	1 (20%)	0 (0%)
	Non‐collaboration (−2)	0 (0%)	0 (0%)
	Minimal collaboration (−1)	0 (0%)	0 (0%)
	Acceptable collaboration (0)	3 (60%)	1 (20%)
	Great collaboration (+1)	0 (0%)	1 (20%)
	Ideal collaboration (+2)	1 (20%)	3 (60%)
Patient/Partner Governance	What statement best describes the nature of collaboration related to meaningful decision‐making between groups?		
	Don't know or not applicable	0 (0%)	0 (0%)
	Non‐collaboration (−2)	0 (0%)	0 (0%)
	Minimal collaboration (−1)	0 (0%)	0 (0%)
	Acceptable collaboration (0)	1 (20%)	0 (0%)
	Great collaboration (+1)	2 (40%)	2 (40%)
	Ideal collaboration (+2)	2 (40%)	3 (60%)
	What statement best describes the nature of collaboration related to accountability between groups?		
	Don't know or not applicable	1 (20%)	1 (20%)
	Non‐collaboration (−2)	0 (0%)	0 (0%)
	Minimal collaboration (−1)	1 (20%)	0 (0%)
	Acceptable collaboration (0)	1 (20%)	0 (0%)
	Great collaboration (+1)	2 (40%)	3 (60%)
	Ideal collaboration (+2)	0 (0%)	1 (20%)
Integration Into the Research Process	What statement best describes the nature of collaboration related to hypothesis generation?		
	Don't know or not applicable	0 (0%)	1 (20%)
	Non‐collaboration (−2)	0 (0%)	0 (0%)
	Minimal collaboration (−1)	0 (0%)	0 (0%)
	Acceptable collaboration (0)	1 (20%)	0 (0%)
	Great collaboration (+1)	1 (20%)	0 (0%)
	Ideal collaboration (+2)	3 (60%)	4 (80%)
	What statement best describes the nature of collaboration related to study design?		
	Don't know or not applicable	0 (0%)	0 (0%)
	Non‐collaboration (−2)	0 (0%)	0 (0%)
	Minimal collaboration (−1)	0 (0%)	0 (0%)
	Acceptable collaboration (0)	0 (0%)	0 (0%)
	Great collaboration (+1)	4 (80%)	0 (0%)
	Ideal collaboration (+2)	1 (20%)	5 (100%)
	What statement best describes the nature of collaboration related to analysis?		
	Don't know or not applicable	0 (0%)	0 (0%)
	Non‐collaboration (−2)	0 (0%)	0 (0%)
	Minimal collaboration (−1)	0 (0%)	0 (0%)
	Acceptable collaboration (0)	1 (20%)	0 (0%)
	Great collaboration (+1)	1 (20%)	1 (20%)
	Ideal collaboration (+2)	3 (60%)	4 (60%)
	What statement best describes the nature of collaboration related to publication?		
	Don't know or not applicable	0 (0%)	0 (0%)
	Non‐collaboration (−2)	0 (0%)	0 (0%)
	Minimal collaboration (−1)	0 (0%)	0 (0%)
	Acceptable collaboration (0)	0 (0%)	0 (0%)
	Great collaboration (+1)	2 (40%)	2 (40%)
	Ideal collaboration (+2)	3 (60%)	3 (60%)
	What statement best describes the nature of collaboration related to attribution?		
	Don't know or not applicable	0 (0%)	0 (0%)
	Non‐collaboration (−2)	0 (0%)	0 (0%)
	Minimal collaboration (−1)	0 (0%)	0 (0%)
	Acceptable collaboration (0)	0 (0%)	0 (0%)
	Great collaboration (+1)	2 (40%)	0 (0%)
	Ideal collaboration (+2)	3 (60%)	5 (100%)
Research Organisation Readiness	What statement best describes the nature of collaboration related to the recognition of biases?		
	Don't know or not applicable	0 (0%)	0 (0%)
	Non‐collaboration (−2)	0 (0%)	0 (0%)
	Minimal collaboration (−1)	0 (0%)	0 (0%)
	Acceptable collaboration (0)	1 (20%)	0 (0%)
	Great collaboration (+1)	2 (40%)	3 (60%)
	Ideal collaboration (+2)	2 (40%)	2 (40%)
	What statement best describes the nature of collaboration related to the collaboration process?		
	Don't know or not applicable	0 (0%)	0 (0%)
	Non‐collaboration (−2)	0 (0%)	0 (0%)
	Minimal collaboration (−1)	0 (0%)	0 (0%)
	Acceptable collaboration (0)	2 (40%)	0 (0%)
	Great collaboration (+1)	1 (20%)	2 (40%)
	Ideal collaboration (+2)	2 (40%)	3 (60%)
	What statement best describes the nature of collaboration related to knowledge in the disease subject?		
	Don't know or not applicable	0 (0%)	0 (0%)
	Non‐collaboration (−2)	0 (0%)	0 (0%)
	Minimal collaboration (−1)	0 (0%)	0 (0%)
	Acceptable collaboration (0)	0 (0%)	0 (0%)
	Great collaboration (+1)	2 (40%)	0 (0%)
	Ideal collaboration (+2)	3 (60%)	5 (100%)

Comments from team members identified strengths and limitations in our approach, highlighting ways to consider improving our process moving forward. Strengths included establishing clear and simple processes for payment of community partners with honoraria; clear communications at a frequency to keep everyone updated but not be burdensome; arranging meetings at mutually convenient times; and providing plenty of notice when work was requested. Examples of areas for improvement in our approach included explicitly establishing terms of reference or culture at the foundation of our study; rules of engagement, and embedding trauma‐informed practices at the outset of a project with community partners; highlighting the need for more concise and clear email communication; and further establishing diversity of representation of the patient population on the team.

When asked about the usefulness of the Scorecards for eliciting perceptions on patient collaborative research efforts, the median score (range −2 to +2) was +1 among researchers and +2 among patient partners. The median for usefulness of the Scorecards for fostering collaborative efforts in research was +1 among researchers and +2 among patient partners. See Table 2 for details. Recommendations for how the Scorecards could be used to enhance collaborative engagement included: new researchers using the Scorecards to guide engagement planning; research team leads educating and preparing their teams for partnership with people with lived experiences; all members of a research team establishing a common language and understanding patient engagement; and funders evaluating patient‐engagement plans for proposed research projects.

**Table 2:** Usefulness of PLRC Scorecards.

Usefulness of PLRC Scorecards	Researchers (*n* = 5) *N* (%)	Patient partners (*n* = 5) *N* (%)
How useful do you think the Scorecards are as a tool to elicit perceptions on patient collaborative research efforts?		
Don't know or not applicable	0 (0%)	1 (20%)
Not at all useful (−2)	0 (0%)	0 (0%)
Somewhat useful (−1)	0 (0%)	0 (0%)
Neutral (0)	0 (0%)	1 (20%)
Very useful (+1)	5 (100%)	0 (0%)
Extremely useful (+2)	0 (0%)	3 (60%)
How useful do you think the Scorecards are as a tool to foster collaborative efforts in research?		
Don't know or not applicable	0 (0%)	1 (20%)
Not at all useful (−2)	0 (0%)	0 (0%)
Somewhat useful (−1)	0 (0%)	1 (20%)
Neutral (0)	0 (0%)	0 (0%)
Very useful (+1)	5 (100%)	0 (0%)
Extremely useful (+2)	0 (0%)	3 (60%)

### 3.2 Guided Discussion—Reflections on Principles and Impact of Community Engagement

Five team members with lived experiences (community partners), representing four community support groups and organisations, met with M.O.H. to discuss our experiences of engagement in the Long COVID and Episodic Disability Study and its impact.

While we used the 4PI framework to guide the discussion, the main focus was on the (1) principles and (2) impact sections. We describe the results of our discussion using the category headings below. We concentrate on these two domains in an attempt to uncover a deeper elucidation of the elements of partnership working which lead to meaningful engagement.

#### 3.2.1 Principles

Community partners discussed the tacit principles exhibited by the entire team throughout the research process. At the outset of the study, we never explicitly discussed what principles we would work with, but it was apparent which ones were threaded through our working relationship. The community partners identified the following six principles.

##### 3.2.1.1 No Hierarchy

While the Co‐PIs and researchers gave strong leadership, there was a flattened hierarchy and equality of voices.

##### 3.2.1.2 Sharing of Power

Decision‐making was done by consensus; there were no situations in which any decisions were imposed by the researchers.

##### 3.2.1.3 Consideration of Fluctuating Capacity to Contribute to Engagement

The researchers were unfailing in their regard for the health needs of the community partners and the impact that this had on their ability to attend meetings and do the work of engagement. The researchers offered flexible ways to contribute, offering their own time for email exchanges or phone calls, depending on what was convenient for the community members.

##### 3.2.1.4 Consideration of the Need to Recruit Participants With Varied Demographics and Life Experiences

It was made explicit that targeted recruitment was our priority to ensure that the cohort included diverse voices and that the community partners were essential in achieving this.

##### 3.2.1.5 Warmth, Friendliness and Empathy

The researchers exhibited palpable care and consideration for the community partners in all dealings. Working with people who have fluctuating capacity inevitably entails extra work for the study team, and the community partners were treated with respect and never made to feel like a burden if they were unable to attend meetings or contribute to the work. The researchers would take the time and effort to develop ways of working to make things as easy as possible for the community partners to contribute. This was done in a spirit of cooperation and presented as a standard practice.

##### 3.2.1.6 Genuine Care for the Team as People

Meetings were used as an opportunity to not only progress the work, but to check in on each other's welfare. At the end of each meeting, the team leaders would offer the chance for everyone to give an update on how they were doing and share any reflections on the work. This fostered an atmosphere of camaraderie and care for each other as people as well as colleagues.

These principles and ways of working emerged out of the personalities and working practices of the team leaders, which set a tone of safety and trust. This atmosphere nurtured the situation in which the community partners felt able to make their contributions with no fear of rebuttal. They felt that no question or observation was too silly or irrelevant and knew that if they raised a point, it would be considered and discussed respectfully. It also engendered a sense of group camaraderie as we learned small details about each other's personal situations. We were able to enjoy each other's wins and commiserate when others were going through one of the troughs, which are an inevitable part of an episodic disability. It also gave researchers a continuous insight into the daily lives of the community partners as they dealt with ongoing illness and the personal challenges of Long Covid.

The community partners were aware of, and appreciative of, the kindness and consideration offered to them by the research team. This, in turn, strengthened their own commitment to the study and the shared goals of the research.

#### 3.2.2 Impact

The community partners discussed what they felt was the impact of their engagement in the work. Key areas in which they felt their contributions had manifested themselves were improving the diversity of the sample, injecting reality into the description of the illness, making the research useful and identifying the most useful next steps for the research. These are described in more detail below.

##### 3.2.2.1 Composition of the Study Population

The study population for the interview phase 1 of the study (*n* = 40) was varied and included people of different ages, genders, sexualities and life experiences. This was a result of deliberate and iterative targeted strategies for recruitment. Initially, the promotion of the study recruited white, heterosexual women in middle age from the first wave of the pandemic. The community partners used their positions within their own communities to appeal for more men, younger people, non‐heterosexual people and those who had been infected with later variants of Covid‐19. The resultant sample of participants included more people who had not been hospitalised with Covid. This was considered important at a time when the medical academic literature was dominated by post‐hospitalisation studies. The position of community partners on the team meant they could reach those who had had no contact with hospital services during their Covid and Long Covid experiences.

##### 3.2.2.2 Describing the Reality

The community partners felt that no previous studies had described the actual realities of living with Long Covid. The elucidation of the episodic and fluctuating nature of the illness was described as more accurate and useful than studies in which recovery was suggested to be a linear process. It was important for community partners that post‐exertional malaise (PEM) was described as largely absent from the narrative elsewhere. This study highlighted the previously overlooked aspects of life with Long Covid, shining light upon a previously invisible illness.

##### 3.2.2.3 Making It Useful

Providing an accurate, real description of the realities of Long Covid meant that the results of this study could be practically useful to individuals who could then take the manuscript to show their doctors to say ‘look this is what it's like for me’ [39]. Because the episodic nature of Long Covid was felt to be missing in existing literature and descriptions of Long Covid, it has been difficult to express to others what the lived experience is like.

##### 3.2.2.4 Influencing the Next Stage of the Research

The findings from the interviews in phase 1 of the study directly informed the measurement phase 2 of the study, to include a PROM. This PROM would differ from existing ones, which have been proposed for Long Covid, in that it would have an element to capture the episodic nature of the illness. This idea arose organically from discussions during the course of the original study and was felt to be important for us to continue to work together to co‐develop the questionnaire based on data from the lived experiences in the phase 1 interviews.

## 4 Discussion

Results from this study describe our process and reflections engaging in an international community‐engaged research study. In our consultation with team members and reflections from the Scorecards, responses were similar for team members who identified as researchers and community partners. This was reassuring as it demonstrated alignment in our process and interpretations of our collaborations as a team.

This study highlights experiences and reflections of engaging in a research study that involves researching a concept (episodic disability) of which members of the team are experiencing first hand. This raised the importance of recognising the impact of this study and how hearing about episodic disability may add considerable burden to team members who were similarly experiencing health challenges. Flexibility, support and acknowledgement of the emotional burden that comes from analysing data from participants who represent your personal experiences within a target population are essential. Establishing terms of reference and values and principles, embedded within a supportive process that encompasses trauma‐informed approaches to co‐create a safe, inclusive and mutually respectful research environment throughout, is vital for community‐engaged research. These recommendations align with earlier work on PPI in HIV research, highlighting the importance of PPI, while highlighting parallel challenges faced with a lack of resources for PPI, and disparate power dynamics and expectations between different interest holder groups [8]. Members of the Long COVID and Episodic Disability Study team are leaders in the field of citizen scientists, setting research priorities that are relevant and meaningful to those living with the condition [25].

Systemic factors which can mitigate against good‐quality patient engagement include short‐term research contracts, constrained research timetables and institutional hierarchies. We were fortunate in our collaboration that none of those problems arose or were brought to bear on the patient partners. Where such situations do arise, research teams need to make extra efforts to mitigate their effects on patient partners.

To our knowledge, this is the first exemplar to explore the utility of the newly developed Patient‐Led Scorecards for the planning and implementation of community‐engaged research [53]. These tools can be helpful in fostering early discussion in the foundational stages of research conceptualisation and design. Trust, effective communication and power sharing are key components of a successful working relationship with community and patient partners [33, 57]. To avoid tokenism and move towards genuine co‐production, it is important for researchers to share power with patient partners, but this is not always achieved in practice [58]. There may be contexts where it is unrealistic to expect patient partners to contribute equally across a research study, as different levels of expertise or capacity will exist across team members. Nevertheless, recognising areas of expertise and having a foundation of trust and transparency in the process will enable parts of a study that may be more researcher‐led and patient/partner informed or consulted, whereas other aspects, such as recruitment or knowledge translation, may be more patient‐led. To create the conditions for a trusting working relationship, strong principles underpinning the work should exist. Much of the research activity focuses on processes and methodologies, but community engagement is a human endeavour and is best achieved not necessarily by following a fixed set of instructions, but rather by reflecting on our relationships. While processes are important to enable and facilitate partnership working, patient engagement is, at its root, a principles‐based endeavour, rather than purely process‐based. The difference between tokenistic and meaningful patient engagement lies not in what things are done, but in how they are done.

### 4.1 Recommendations

Our study featured many of the practical components shown to be important in community engagement, which can be found in most guidance documents. In Table 3, we propose a series of recommendations and considerations focused on practices that underpin meaningful community engagement and lead to impact and a positive experience for all concerned. These practices speak to the embodiment and manifestation of principles that pave the way for the sharing of power across a study team. Unique features of these recommendations are that they are directed to both persons with lived experiences as well as researchers, and they were derived from the perspectives of navigating the research landscape while living with a chronic and episodic condition such as Long Covid, which can be unique to other studies focused on different conditions. For these recommendations to be successfully implemented, community members must be sufficiently empowered to feel comfortable challenging the status quo of a research process. This makes it all the more important that research teams make efforts to ensure that conditions exist such that community partners feel enabled and supported to challenge the process where they feel it necessary.

**Table 3:** Recommendations for researchers and for community partners.

Recommendations for researchers and for community partners	
**Recommendations for researchers**	
Establish your underlying principles	Consider the principles which will underpin your interactions with community partners, as this will set the tone for the partnership. You may want to use an established framework such as the 4PI Framework, Patient‐Led Research Scorecards or the UK Standards for Involvement as a guide.
Allow enough time	Anticipate that community engagement with people with a health condition, especially an energy‐limiting one, will be an evolving process where you may not be able to control the research process as much as you are used to doing. Expect a certain amount of timeline slippage and having to ‘go with the flow’.
Leadership Style	The principal investigator/s need to lead, but it must be in a collaborative style rather than ‘command and control’. The process of decision‐making should be transparently collaborative.
Continuous reflection	Continuous reflection will be needed to make sure that processes are still working and that everyone is happy with how the collaboration is progressing. An evaluation framework, such as the Patient‐Led Research Scorecards, can be used as a tool to monitor periodically.
Establish and nurture trust	Trust is essential and that can only arise if everyone feels safe and supported to contribute. Anything which can be done to ensure that all team members are facilitated to remain involved should be done. Practically speaking this can include regularly checking in on everyone's welfare, whether in meetings or offline.
Fairness of recognition	Offer co‐authorship on manuscripts in a meaningful way, allowing time for community partners to read and digest them and be able to offer informed comments. This may well entail more iterations than you are used to.
Care for community partners	There is a balance to be struck between taking care of community members' health while allowing them to decide how much energy to give. It was not uncommon for community partners to join meetings while lying in bed, as they didn't have the energy to sit up. While there may be a temptation to say that they shouldn't continue to attend as they are clearly ill, as long as it is understood that there is no pressure to remain and they can leave the meeting whenever they like, it is preferable that it is the community member's decision to attend or not.
Update on progress	Community members want to feel as though we are all in this together working to achieve a common goal. They are giving their precious energy and the most important thing to them is that it means something and will have impact. Keeping everyone updated as to progress and how their inputs are shaping the study is essential to maintain motivation.
**Recommendations for community partners**	
Ask who is your point of contact	It should be clear who the point of contact is for study‐related administration tasks such as sorting out honoraria payments or joining meetings. There should be one or two people in the study team who you can ask to help you with these tasks.
Ask for accommodations	Don't hesitate to ask for accommodations and adjustments where you need them if they haven't already been offered. Researchers may not have experience working with people with a chronic condition and may not know what accommodations you need.
Ask for information in a way that you can understand	Engagement in research may be challenging, but it shouldn't feel like taking an exam. If you're finding it too difficult to understand what's going on, it may be that the information needs to be presented in a different way. Raise this with the research team and ask for them to explain things in a different way that you can follow. Other community partners may be grateful, as it's likely that if you're finding it difficult, others are too.
Share feedback	Interactions with the research team should feel like a positive experience. If you are feeling apprehensive or uncomfortable, then consider sharing this feedback with the study team to discuss your concerns. You may want to speak to other community partners about it too, as others may be feeling the same.

### 4.2 Strengths and Limitations

This study was derived from an exemplar involving an international study involving a multidisciplinary team including persons with lived experiences of Long Covid, clinicians and researchers who were involved in the study since the conceptualisation of the study objectives and acquisition of funding. This study was derived from a longstanding international collaboration in HIV, disability and rehabilitation (D.B. and K.K.O.); the field of HIV and community‐based research was instrumental in informing the approaches among the research team. This study builds on the foundational literature of community‐based research and the GIPA/MEPA and the disability community principles of participation, of ‘nothing about us without us’ [7].

Nevertheless, our team and the Long COVID and Episodic Disability Study are situated in high‐income countries, with limited diversity, raising a query as to whether these lessons learned are transferable to other contexts.

This study was the first to use the Patient‐Led Scorecards to formally evaluate community engagement in the context of a Long Covid research study. Responses to the Patient‐Led Research Scorecard questionnaire are descriptive in nature. We developed the Patient‐Led Research questionnaire for the purposes of our consultation, whereby items in the questionnaire were directly derived from the Patient‐Led Research Scorecards and reviewed by members of PLRC on the team involved in the creation of the Scorecards (L.M. and H.W.). Nevertheless, the measurement properties of the tool are unknown.

While our consultation included a community‐led discussion with patient partners on the team, followed by an anonymous questionnaire, it is possible that team members may have felt reluctant to voice concerns or limitations in the team process, as the study is ongoing. The combination of lead author (M.O.H.) expertise in PPI with the co‐PIs' (K.K.O. and D.A.B.) experiences with community involvement in their research and clinical practices in the context of HIV, disability and rehabilitation meant we were able to bring a compilation of experiences and lessons learned to this study. Hence, we feel that the findings accurately reflect the strengths as well as considerations for improvement for engaging in this Long COVID and Episodic Disability Study. However, we acknowledge that the positive results of our process evaluation are a reflection of the community leaders living with Long Covid on this team who had strong coalitions and embedded academic expertise prior to the start of this study; otherwise, this dynamic process may not have been so fruitful or could have been limited. It is important to note that patients often lack institutional support that academics have access to; hence, ensuring individual comfort and empowerment, in conjunction with helping to support the communities behind the patient partners, is critical to this process. Finally, this manuscript is derived primarily from our process in phase 1 (interview) of the Long COVID and Episodic Disability Study; phase 2 (web‐based questionnaire with the EDQ and Long COVID Supplement) is still underway. We are 4 years into this study since our acquisition of funding in 2021. We continue to build this research programme and collaborate on other research related to Long Covid, disability and rehabilitation. Hence, our process and lessons learned will evolve as a team as the study continues over time.

## 5 Conclusions

The Long COVID and Episodic Disability Study was a community‐engaged research study involving a team of over 20 persons with lived experiences, researchers and clinicians in Canada, Ireland, the United Kingdom and the United States. All aspects of the study were guided by the engagement of persons with lived experiences as members of the core team. Our process involved practical strategies to facilitate the engagement of team members experiencing episodic disability. Our community‐engaged approach enhanced the quality and relevance of the study to the community while highlighting areas to heighten meaningful engagement throughout. This study builds on foundational community‐based research principles of patient‐oriented research. Recommendations derived from our experiences can be used by other research teams conducting community‐engaged patient‐oriented research.

## Author Contributions

**Margaret E. O'Hara:** conceptualisation, methodology, data curation, writing – review and editing, writing – original draft, investigation, funding acquisition, validation, visualisation, formal analysis, project administration. **Kiera McDuff:** conceptualisation, methodology, investigation, validation, funding acquisition, writing – original draft, visualisation, writing – review and editing, formal analysis, project administration, data curation. **Hannah Wei:** conceptualisation, writing – review and editing, methodology, funding acquisition, investigation. **Lisa McCorkell:** conceptualisation, methodology, investigation, funding acquisition, writing – review and editing. **Catherine Thomson:** conceptualisation, methodology, investigation, funding acquisition, writing – review and editing. **Mary Kelly:** conceptualisation, investigation, funding acquisition, methodology, writing – review and editing. **Susie Goulding:** conceptualisation, investigation, funding acquisition, writing – review and editing, methodology. **Imelda O'Donovan:** conceptualisation, methodology, investigation, funding acquisition, writing – review and editing. **Sarah O'Connell:** conceptualisation, methodology, investigation, funding acquisition, writing – review and editing. **Ruth Stokes:** conceptualisation, investigation, funding acquisition, methodology, writing – review and editing. **Nisa Malli:** methodology, conceptualisation, investigation, funding acquisition, writing – review and editing. **Natalie St Clair‐Sullivan:** conceptualisation, investigation, funding acquisition, methodology, writing – review and editing. **Soo Chan Carusone:** conceptualisation, investigation, funding acquisition, methodology, writing – review and editing. **Angela M. Cheung:** conceptualisation, methodology, investigation, funding acquisition, writing – review and editing. **Kristine M. Erlandson:** conceptualisation, investigation, funding acquisition, methodology, writing – review and editing. **Ciaran Bannan:** conceptualisation, investigation, funding acquisition, methodology, writing – review and editing. **Liam Townsend:** conceptualisation, investigation, funding acquisition, methodology, writing – review and editing. **Colm Bergin:** conceptualisation, investigation, funding acquisition, methodology, writing – review and editing. **Jaime H. Vera:** conceptualisation, investigation, funding acquisition, methodology, writing – review and editing. **Richard Harding:** conceptualisation, investigation, funding acquisition, methodology, writing – review and editing. **Lisa Avery:** conceptualisation, investigation, funding acquisition, methodology, writing – review and editing. **Darren A. Brown:** conceptualisation, investigation, funding acquisition, methodology, writing – review and editing. **Kelly K. O'Brien:** conceptualisation, investigation, funding acquisition, methodology, writing – review and editing, writing – original draft, validation, visualisation, software, formal analysis, project administration, data curation, supervision, resources.

## Conflicts of Interest

The authors declare no conflicts of interest.

## Ethics Statement

The Long COVID and Episodic Disability Study was approved by the Health Sciences Research Ethics Board at the University of Toronto (Protocol #41749) and the Saint James Hospital (SJH)/Tallaght University Hospital (TUH) Joint Research Ethics Committee (2024‐Mar‐34453445).

## Supporting information

Supplemental File 1 ‐ Community Engagement in Long COVID Research: Process, Evaluation and Recommendations from the Long COVID and Episodic Disability Study.

Supplemental File 2 ‐ Patient‐Led Research Scorecard Questionnaire and the Long COVID and Episodic Disability Study.

## Data Availability

The data that support the findings of this study are available from the corresponding author upon reasonable request. All relevant data are included in the manuscript and its Supporting Information files.
