# Supplementary material for: Community Engagement in Long Covid Research: Process, Evaluation and Recommendations From the Long COVID and Episodic Disability Study
Source: Health Expect. 2025 Aug 10;28(4):e70365. doi: 10.1111/hex.70365 (PMC12335852; doi:10.1111/hex.70365)
Supplement: Supplementary file 1 — Supplemental File 1 ‐ Community Engagement in Long COVID Research: Process, Evaluation and Recommendations from the Long COVID and Episodic Disability Study. [file HEX-28-e70365-s002.pdf]

Discussion Guide

Guided Discussion with the 4PI Framework

Patient Involvement in Episodic Disability in Long Covid Research Study

4PI stands for **'Principles; Purpose; Presence; Process; Impact'**; it is a framework to help codify ways of working between groups of people with a common purpose or project.

The 4PI framework helps working groups to think through their *purpose*, *how* they will work together and *how* they will evaluate whether they have achieved their purpose. It is proposed that we use this as a framework to achieve the objectives that we are already working towards.

Principles:

Suggestions as a starter for 90-min discussion:

- **No Hierarchy**
- **Sharing of Power**
- **Warmth**
- **Friendliness**
- **Empathy**

Guided Questions included:

1) Please reflect on how you worked together throughout the study according to each of the categories of the 4PI.
    a. No Hierarchy
    b. Sharing of Power
    c. Warmth
    d. Friendliness
    e. Empathy
2) What has gone well during the process?
3) What has been a challenge or what could be done differently?
4) What are some considerations for future patient engagement in Long COVID research?

We acknowledge the National Survivor User Network for the 4PI Framework
