# Supplementary material for: Community Engagement in Long Covid Research: Process, Evaluation and Recommendations From the Long COVID and Episodic Disability Study
Source: Health Expect. 2025 Aug 10;28(4):e70365. doi: 10.1111/hex.70365 (PMC12335852; doi:10.1111/hex.70365)
Supplement: Supplementary file 2 — Supplemental File 2 ‐ Patient‐Led Research Scorecard Questionnaire and the Long COVID and Episodic Disability Study. [file HEX-28-e70365-s001.pdf]

# Patient-Led Research Scorecard Questionnaire
# Community Engagement in the Long COVID and Episodic Disability Study

## What is the aim of this questionnaire?

To glean perceptions on patient collaborative research efforts within the Long COVID and Episodic Disability Study.

## How do you complete this questionnaire?

Using the PLRC Scorecards as a foundation, members of the team are asked to collectively (as an organization) reflect and discuss their experiences with the study. The questionnaire is broken into the following 4 areas of the scorecards:

- Patient / Partner Governance
- Integration into Research Process
- Patient Burden
- Research Organization Readiness

As a community organization or network, please **collectively discuss and complete** the following questionnaire, regarding your experiences and reflections with the Long COVID and Episodic Disability study (1 response per network / organization). Please feel free to add comments in the open text boxes.

To complete, please refer to the PLRC scorecards for descriptions for each level of collaboration, from non-collaboration (-2) to ideal collaboration (2). https://patientresearchcovid19.com/storage/2023/02/Patient-Led-Research-Scorecards.pdf. **Tip**: It is helpful to have the scorecards either printed out or on a second computer screen so that you view the scorecards while completing the questionnaire.

## How will this questionnaire be used?

Responses will be used to inform the manuscript discussing our reflections on the community engaged process with the Long COVID and Episodic Disability Study.

## Acknowledgement

This questionnaire is derived from the Patient-Led Research Scorecards, developed to evaluate how patient group and research partner collaboration in a research study.

Patient-Led Research Scorecards: The Promise of Patient-Led Research Integration into Clinical Registries and Research. Patient-Led Research Collaborative & Council of Medical Speciality Societies. 2023. https://patientresearchcovid19.com/storage/2023/02/Patient-Led-Research-Scorecards.pdf

# Patient-Led Research Scorecard Questionnaire

*Role on the Team / Process for Completion*

**What best describes your role on the Long COVID and Episodic Disability Study Team / process for completing the questionnaire?**

*Team-Based Completion*

□ Group of persons with lived experiences representing a community organization or network

*Individual Completion*

□ Person with Lived Experiences representing a community organization or network
□ Researcher
□ Clinician
□ Research Coordinator
□ Trainee
□ Person with Lived Experiences

## Patient-Led Research Scorecard Questionnaire

For each of the following statements, please rate your perception of the following from, -2 (non-collaboration) to 2 (ideal collaboration). Please add further comments in the open text box. *Please see the Patient-Led Scorecards for the description of each category of collaboration.*

**Section 1 - Patient Burden:** This section evaluates the degree to which patient burden and associated trauma is addressed, including accommodating patients who are dealing with illness and symptoms, compensation for patients' time and skills.

**For each component, what statement best describes the nature of collaboration as it relates to patient burden?**

| Item # | Component | Statement of Collaboration – see Score Cards for description of each | | | | | |
|---|---|---|---|---|---|---|---|
| 1 | **Accessible Engagement** | Non-Collaboration ☐ (-2) | Minimal Collaboration ☐ (-1) | Acceptable Collaboration ☐ (0) | Great Collaboration ☐ (1) | Ideal Collaboration ☐ (2) | Don't Know or Not Applicable ☐ |
| 2 | **Trauma-Informed Practices** | Non-Collaboration ☐ (-2) | Minimal Collaboration ☐ (-1) | Acceptable Collaboration ☐ (0) | Great Collaboration ☐ (1) | Ideal Collaboration ☐ (2) | Don't Know or Not Applicable ☐ |
| 3 | **Responsiveness to Patients** | Non-Collaboration ☐ (-2) | Minimal Collaboration ☐ (-1) | Acceptable Collaboration ☐ (0) | Great Collaboration ☐ (1) | Ideal Collaboration ☐ (2) | Don't Know or Not Applicable ☐ |
| 4 | **Compensation** | Non-Collaboration ☐ (-2) | Minimal Collaboration ☐ (-1) | Acceptable Collaboration ☐ (0) | Great Collaboration ☐ (1) | Ideal Collaboration ☐ (2) | Don't Know or Not Applicable ☐ |
| **Comments** | | | | | | | |

**Section 2 – Patient / Partner Governance:** This section evaluates the degree to which decision-making power and governance is shared between patient groups and partner groups

**For each component, what statement best describes the nature of collaboration as it relates to patient/partner governance?**

| Item # | Component | Statement of Collaboration – see Score Cards for description of each | | | | | |
|---|---|---|---|---|---|---|---|
| 5 | **Meaningful Decision-making between groups** | Non-Collaboration ☐ (-2) | Minimal Collaboration ☐ (-1) | Acceptable Collaboration ☐ (0) | Great Collaboration ☐ (1) | Ideal Collaboration ☐ (2) | Don't Know or Not Applicable ☐ |
| 6 | **Accountability between groups** | Non-Collaboration ☐ (-2) | Minimal Collaboration ☐ (-1) | Acceptable Collaboration ☐ (0) | Great Collaboration ☐ (1) | Ideal Collaboration ☐ (2) | Don't Know or Not Applicable ☐ |
| **Comments** | | | | | | | |

**Section 3 – Integration into Research Process**: This section evaluates the degree to which patients are involved in every phase of the research process and key committees, including study design, protocols, trial inclusion, analysis, and reporting.

**For each component, what statement best describes the nature of collaboration as it relates to integration into the research process?**

| Item # | Component | Statement of Collaboration – see Score Cards for description of each | | | | | |
|---|---|---|---|---|---|---|---|
| 7 | **Hypothesis Generation** | Non-Collaboration ☐ (-2) | Minimal Collaboration ☐ (-1) | Acceptable Collaboration ☐ (0) | Great Collaboration ☐ (1) | Ideal Collaboration ☐ (2) | Don't Know or Not Applicable ☐ |
| 8 | **Study Design** | Non-Collaboration ☐ (-2) | Minimal Collaboration ☐ (-1) | Acceptable Collaboration ☐ (0) | Great Collaboration ☐ (1) | Ideal Collaboration ☐ (2) | Don't Know or Not Applicable ☐ |
| 9 | **Analysis** | Non-Collaboration ☐ (-2) | Minimal Collaboration ☐ (-1) | Acceptable Collaboration ☐ (0) | Great Collaboration ☐ (1) | Ideal Collaboration ☐ (2) | Don't Know or Not Applicable ☐ |
| 10 | **Publication** | Non-Collaboration ☐ (-2) | Minimal Collaboration ☐ (-1) | Acceptable Collaboration ☐ (0) | Great Collaboration ☐ (1) | Ideal Collaboration ☐ (2) | Don't Know or Not Applicable ☐ |
| 11 | **Attribution** | Non-Collaboration ☐ (-2) | Minimal Collaboration ☐ (-1) | Acceptable Collaboration ☐ (0) | Great Collaboration ☐ (1) | Ideal Collaboration ☐ (2) | Don't Know or Not Applicable ☐ |

| Item # | Component | Statement of Collaboration – see Score Cards for description of each | | | | | |
|---|---|---|---|---|---|---|---|
| Comments | | | | | | | |

**Section 4 – Research Organization Readiness**: This section evaluates the ability of the research organization to engage in meaningful patient partnership. This readiness assessment allows patients to discern the research organization's level of collaboration and willingness to share control.

**For each component, what statement best describes the nature of collaboration as it relates to research organization readiness?**

| Item # | Component | Statement of Collaboration – see Score Cards for description of each | | | | | |
|---|---|---|---|---|---|---|---|
| 12 | **Recognition of Biases** | Non-Collaboration ☐ (-2) | Minimal Collaboration ☐ (-1) | Acceptable Collaboration ☐ (0) | Great Collaboration ☐ (1) | Ideal Collaboration ☐ (2) | Don't Know or Not Applicable ☐ |
| 13 | **Collaboration Process** | Non-Collaboration ☐ (-2) | Minimal Collaboration ☐ (-1) | Acceptable Collaboration ☐ (0) | Great Collaboration ☐ (1) | Ideal Collaboration ☐ (2) | Don't Know or Not Applicable ☐ |
| 14 | **Knowledge in Disease Subject** | Non-Collaboration ☐ (-2) | Minimal Collaboration ☐ (-1) | Acceptable Collaboration ☐ (0) | Great Collaboration ☐ (1) | Ideal Collaboration ☐ (2) | Don't Know or Not Applicable ☐ |
| **Comments** | | | | | | | |

## Final Questions – Utility of the Scorecards

| Item # | Question | Response Options | | | | | |
|---|---|---|---|---|---|---|---|
| 15 | **How useful do you think the Scorecards are as a tool to elicit perceptions on patient collaborative research efforts?** | Not at all useful<br>☐<br>(-2) | Somewhat useful<br>☐<br>(-1) | Neutral<br>☐<br>(0) | Very useful<br>☐<br>(1) | Extremely useful<br>☐<br>(2) | Don't Know or Not Applicable<br>☐ |
| **Comments** | | | | | | | |
| 16 | **How useful are the Scorecards as a tool to foster collaborative efforts in research?** | Not at all useful<br>☐<br>(-2) | Somewhat useful<br>☐<br>(-1) | Neutral<br>☐<br>(0) | Very useful<br>☐<br>(1) | Extremely useful<br>☐<br>(2) | Don't Know or Not Applicable<br>☐ |
| **Comments** | | | | | | | |
| 17 | **Do you have any recommendations for how the Patient-Led Scorecards may be used to enhance collaborative engagement in research?** | Yes<br>☐<br>(1) | No<br>☐<br>(0) | Don't Know<br>☐ | | | |
| **If Yes, Please Describe:** | | | | | | | |
